# Prevalence of low bone mineral density among HIV patients on long-term suppressive antiretroviral therapy in resource limited setting of western India

**DOI:** 10.7448/IAS.17.4.19567

**Published:** 2014-11-02

**Authors:** Ameet Dravid, Milind Kulkarni, Amit Borkar, Sachin Dhande

**Affiliations:** 1 HIV Medicine, Ruby Hall Clinic, Pune, India

## Abstract

**Introduction:**

Bone mineral density (BMD) assessment in HIV patients is sparsely done in resource limited settings.

**Materials and Methods:**

We conducted a cross-sectional study of BMD amongst HIV patients following up in our clinic from 1 June to 1 December 2013 by performing dual-energy X-ray absorptiometry scan (Lunar Prodigy Advanced DXA System, GE Healthcare) of lumbar spine and hip. Patients on long term (≥12 months), virologically suppressive antiretroviral therapy (ART) were included. Patients who were ART naïve were included as control population. Virologic failures were excluded. Low BMD was defined by WHO T-score criteria (normal: T score ≥−1;osteopenia: T score between −1 and −2.5 SD; osteoporosis: T score ≤−2.5 SD). Baseline risk factors associated with low BMD like age, low BMI, lipoatrophy, diabetes mellitus, current smoking, current alcohol intake, steroid exposure and menopause were recorded. ART-related factors associated with low BMD like ART duration, exposure to tenofovir and exposure to protease inhibitors (PI) were studied.

**Results:**

A total of 536 patients (66% males, 496 ART experienced and 40 ART naïve) were included in this analysis. Median age was 42 years, mean BMI 23.35 kg/m^2^ and median CD4 count 146 cells/mm^3^. All ART experienced patients had plasma viral load<400 copies/ml.

Prevalence of low BMD amongst ART naive and ART experienced patients was 67% (osteopenia: 70.4%, osteoporosis: 29.6%) and 80.4% (osteopenia: 63.4%, osteoporosis: 36.6%), respectively (p=0.05). Mean T scores at lumbar spine and hip for ART naive and ART experienced patients were −1.37 and −0.9 versus −1.56 and −1.48 (p=0.05), respectively. Age, low BMI, current smoking, menopause, baseline CD4 count and exposure to ART were factors significantly associated with low overall BMD on univariate regression analysis. On multivariable logistic regression analysis age (p<0.001), low BMI (p<0.001), current smoking (0.05) and menopause (0.03) were associated with low BMD. BMI decrease of 1 kg/m^2^ and baseline CD4 decline of 50 cells/mm^3^ lead to 14% and 4% increased probability of low BMD, respectively. Choice of antiretroviral use (tenofovir vs non-tenofovir, PI vs No PI) did not influence loss of BMD.

**Conclusions:**

Extremely high prevalence of accelerated BMD loss amongst ART naïve and ART experienced patients in our cohort is a matter of deep concern due to its association with pathological fractures. Bone mineral loss was seen irrespective of ART used. Association of low BMD with low baseline CD4 count strengthens the case for early ART.

**Table 1:** Baseline characteristic of patients with normal and low BMD

Characteristic	Overall	Normal BMD	Low BMD	P value
Age (median, IQR)	42 (37–48)	39 (35–44)	44 (38–49)	P<0.001
BMI (median, IQR)	23.3 (20.2–26.4)	25.4 (22.2–28.6)	22.8 (19.7–25.8)	P<0.001
Lipoatrophy, n (%)	61 (11%)	8 (7%)	53 (12%)	0.13
Steroid, n (%)	47 (9%)	7 (6%)	40 (9%)	0.32
Smoking, n (%)	26 (5%)	2 (2%)	24 (6%)	0.1
Alcohol, n (%)	56 (10%)	12 (11%)	44 (10%)	0.86
Diabetes, n (%)	26 (5%)	5 (5%)	21 (5%)	0.87
Baseline CD4, median (IQR)	146 (72–131)	179 (76–287)	140 (72–220)	0.046
ART experienced, n (%)	496 (90%)	97 (88%)	399 (94%)	0.05

**Table 2:** Univariate and multivariate regression analysis of risk factors associated with low BMD

Characteristic	Unadjusted OR	95% CI; p value	Adjusted OR	95% CI; p value
Age	1.05	1.02,1.06; p < 0.001	1.06	1.03,1.09; p < 0.001
Female gender	1.01	0.64,1.57; p = 0.97	1.32	0.82,2.16; p = 0.26
BMI	0.89	0.85,0.93; p < 0.001	0.86	0.82,0.91; p < 0.001
Lipoatrophy	1.81	0.83,3.93; p = 0.13		
Steroid	1.52	0.66,3.5; p = 0.32	1.41	0.58,3.4; p = 0.45
Smoking	3.22	0.75,13.85; p = 0.12	4.84	1.01,23.3; p = 0.05
Alcohol	0.94	0.48,1.88; p = 0.86		
Menopause	9.89	2.29,42.8; p = 0.002		
Baseline CD4 count	0.92	0.88,0.97; p = 0.003	0.96	0.9,1.03; p = 0.28
ART exposure	1.98	0.99,3.98; p = 0.05	1.18	0.48,2.87; p = 0.28
TDF exposure	1.18	0.58,2.4; p = 0.65		

